# Lipidomic and metabolomic characterization of a genetically modified mouse model of the early stages of human type 1 diabetes pathogenesis

**DOI:** 10.1007/s11306-015-0889-1

**Published:** 2015-11-17

**Authors:** Anne Julie Overgaard, Jacquelyn M. Weir, David Peter De Souza, Dedreia Tull, Claus Haase, Peter J. Meikle, Flemming Pociot

**Affiliations:** Pediatric Department, Herlev Hospital, Herlev Ringvej 75, 2730 Herlev, Denmark; Baker IDI Heart and Diabetes Institute, Melbourne, Australia; Metabolomics Australia, Bio21 Institute, University of Melbourne, Parkville, Melbourne, Australia; Novo Nordisk A/S, Måløv, Denmark; Department of Biochemistry and Molecular Biology, University of Melbourne, Melbourne, Australia

**Keywords:** Lipidomics, Metabolomics, Type 1 diabetes

## Abstract

The early mechanisms regulating progression towards beta cell failure in type 1 diabetes (T1D) are poorly understood, but it is generally acknowledged that genetic and environmental components are involved. The metabolomic phenotype is sensitive to minor variations in both, and accordingly reflects changes that may lead to the development of T1D. We used two different extraction methods in combination with both liquid- and gas chromatographic techniques coupled to mass spectrometry to profile the metabolites in a transgenic non-diabetes prone C57BL/6 mouse expressing CD154 under the control of the rat insulin promoter (RIP) crossed into the immuno-deficient recombination-activating gene (RAG) knockout (−/−) C57BL/6 mouse, resembling the early stages of human T1D. We hypothesized that alterations in the metabolomic phenotype would characterize the early pathogenesis of T1D, thus metabolomic profiling could provide new insight to the development of T1D. Comparison of the metabolome of the RIP CD154 × RAG^−/−^ mice to RAG^−/−^ mice and C57BL/6 mice revealed alterations of >100 different lipids and metabolites in serum. Low lysophosphatidylcholine levels, accumulation of ceramides as well as methionine deficits were detected in the pre-type 1 diabetic mice. Additionally higher lysophosphatidylinositol levels and low phosphatidylglycerol levels where novel findings in the pre-type 1 diabetic mice. These observations suggest that metabolomic disturbances precede the onset of T1D.

**Electronic supplementary material:**

The online version of this article (doi:10.1007/s11306-015-0889-1) contains supplementary material, which is available to authorized users.

## Introduction

Type 1 diabetes (T1D) is a common chronic illness with increasing prevalence that has serious economic consequences to health care systems (Hex et al. 2012; ADA 2013; IDF 2013). T1D is a complex, multifactorial autoimmune disease in which the insulin-producing pancreatic beta cells are destroyed, leaving the patient dependent on insulin injections for sustaining life, and in risk of premature death due to acute- and late complications (Daneman 2006). The exact mechanisms triggering and regulating progression towards beta cell failure in T1D development are not fully understood. It is generally acknowledged, however, that both genetic and environmental components are involved. The metabolomic phenotype is sensitive to minor variations in both, and may thus reflect changes that lead to the development of T1D. A detailed understanding of the pathogenesis of T1D is a prerequisite for the development of preventive strategies. In particular, the identification of early metabolic modifications is promising in the study of etiological pathways and can potentially lead to the development of biomarkers for beta cell health before clinical symptoms appear, so that preventive strategies to save beta-cells can be initiated. Previous studies have linked metabolomic disturbances to the development of T1D both in humans and in non-obese diabetic (NOD) mice, revealing associations between the immune system, metabolism and the development of T1D (Orešič et al. 2008; Sysi-Aho et al. 2011). However, it remains unclear how the immune system interacts with the metabolome and vice versa in the T1D disease pathogenesis.

In this study, we used a murine transgenic model that was originally designed for the study of the initial events leading to T1D (Haase et al. 2004; Haase and Markholst 2007). The transgenic mice express CD154 (CD40 ligand) under the control of the rat insulin promoter (RIP) to confine the expression to the pancreatic beta cells. In this model, ligation of CD154 with CD40 converts an immature and tolerogenic dendritic cell (DC) to a mature and immunogenic DC (Bennett et al. 1998; Ridge et al. 1998; Schoenberger et al. 1998; Grohmann et al. 2001) leading to insulitis and diabetes development. The mice were crossed into a recombination activating gene (RAG)-deficient background (RAG KO). The RAG genes encodes enzymes crucial for the generation of mature B and T cells (Nishana and Raghavan 2012), and by introducing this genetic modification, the authors showed that T1D development was T- and B cell dependent, based on the observations that the RIPCD154 × RAG^−/−^ mice did not develop diabetes as opposed to their transgene positive litter mates RIPCD154 × RAG^−/+^. Histological examinations of the pancreas of the RIPCD154 × RAG^−/−^ (from here on referred to as RIPCD154 × RAG KO) revealed less destructive cellular infiltrates mainly dominated by CD11c+ cells indicating that the infiltrating cells were local DCs (Haase et al. 2004). Thus, this mouse model was used to study the early phases of the T1D pathogenesis defined as the non-lymphocyte-dependent initial phase (Nerup et al. 1994) characterized by unspecific inflammation of the pancreatic islets (Eisenbarth 1986). In the present study we used this model to characterize the metabolomic changes preceding T1D, without the influence of the adaptive immune system. We compared the RIP CD154 × RAG KO mice to RAG KO mice and C57BL/6 control mice to reveal early metabolomic alterations without the immune component, to elucidate the mechanisms leading to T1D. With the recent advances in high throughput mass spectrometry based technologies, it is now possible to achieve thorough metabolomics investigation of fluids, such as serum, and tissue (Zhang et al. 2014a). We aimed at investigating differences in the serum metabolome using a single phase extraction method combined with a targeted lipidomic approach by liquid chromatography electrospray ionization tandem mass spectrometry (LC ESI–MS/MS) and furthermore, a polar metabolite profiling through gas chromatography mass spectrometry (GC–MS), where the resultant data was analysed using targeted and untargeted data processing methodologies. Using two different mass spectrometry based strategies for in-depth characterization of this murine model revealed metabolomic insight into the disease pathogenesis in the early stages leading to T1D, and identified metabolites with the potential to be developed into future biomarkers of beta cell health.

## Materials and methods

### Animals

Both transgenic (RIP-CD154 × RAG KO (n = 6) and RAG KO (n = 3) on a C57BL/6 background) and control mice (C57BL/6) (n = 10) were bred at Taconic M&B, Ry, Denmark, and genotyped by PCR as previously described (Haase et al. 2004; Haase and Markholst 2007). The animals, all female and 12 weeks old, were housed in polyethylene cages in a temperature, humidity and 12 h day–night rhythm controlled room receiving standard chow. After weighing of the animals, blood samples were collected retro-orbital, without anesthesia, into eppendorf tubes and the animals sacrificed immediately afterwards. The blood samples were left for coagulation and hereafter centrifuged (15 min at 2000×*g* at RT) for the collection of serum. Blood glucose was measured in serum using the Accu-Chek Aviva system by Roche. All animal experiments were conducted according to Danish legislation and approved by the Danish Animal Inspectorate.

### Lipidomics

Samples were randomized and lipids extracted from serum (10 µL) using a single phase chloroform methanol (2:1) method following addition of 15 internal standards (supplementary Table 3), as described in detail previously (Weir et al. 2013). Lipid analysis was performed using an Agilent 1200 liquid chromatography system coupled to an Applied Biosystem API 4000 Q/TRAP mass spectrometer with a turbo-ionspray source and Analyst 1.5 data system. Over 300 species of lipid were analysed including species of: dihydroceramide (dhCer), ceramide (Cer), monohexosylceramide (MHC), dihexosylceramide (DHC), trihexosylceramide (THC), GM3 ganglioside (GM3), sphingomyelin (SM), phosphatidylcholine (PC), alkylphosphatidylcholine (PC(O)), alkenylphosphatidylcholine (plasmalogen, PC(P)), lysophosphatidylcholine (LPC), lysoalkylphosphatidylcholine (lysoplatelet activating factor, LPC(O)), phosphatidylethanolamine (PE), alkylphosphatidylethanolamine (PE(O)), alkenylphosphatidylethanolamine (plasmalogen (PE(P)), phosphatidylinositol (PI), lysophosphatidylinositol (LPI), phosphatidylserine (PS), phosphatidylglycerol (PG), free cholesterol (COH), cholesteryl ester (CE), diacylglycerol (DG) and triacylglycerol (TG) using multiple reaction monitoring (MRM) experiments, described previously (Meikle et al. 2013; Weir et al. 2013). The abbreviations listed here refer to the lipid classes and subclasses, the number of carbons and double bonds will be listed when referring to individual lipid species, such as LPC 22:6 which define a lysophosphatidylcholine containing a fatty acid comprising 22 carbon atoms and six double bonds. Lipids composed of two fatty acids are determined as the sum of the carbons and the double bonds across both fatty acids, e.g. PC 36:4 as described previously (Meikle et al. 2013). Relative concentrations of lipid classes and subclasses were calculated from the sum of the individual lipid species within each class.

### Polar metabolomics

Serum samples and nine pooled (an aliquot of all the samples) quality control samples incorporated into the analysis sequence, were extracted in a 1:3:1 (v/v/v) ratio of chloroform:methanol:water, where 20 µL serum was regarded as the water phase. Briefly, 20 µL chloroform was added to the serum and vortexed to mix, followed by the addition of 60 µL methanol [containing internal standards; ^13^C-sorbitol (16.6 µM) and ^13^C,^15^N-valine (166 µM), Sigma], vortexed, then allowed to incubate on ice for 10 min. Samples were then centrifuged for 5 min at 14000 rpm at 4 °C to pellet precipitated proteins and the supernatant transferred to a fresh tube. Next 40 µL Milli-Q water was added to the supernatant to bring the solvent ratio to 1:3:3 (v/v/v) chloroform:methanol:water, thereby enabling biphasic partitioning of the extract. After vortexing, samples were centrifuged to clearly separate the aqueous (methanol/water) and organic layers (chloroform). 60 µL of the upper aqueous layer containing polar metabolites was taken and evaporated to complete dryness in vacuo. The extraction protocol was modified from the Bligh-Dyer protocol from 1957 (Bligh and Dyer 1959).

Polar metabolites were derivatised online using a Gerstel MPS2 XL autosampler robot (Gerstel, Germany). Samples were first methoxyaminated by the addition of 20 µL methoxyamine (30 mg/mL in pyridine, 2 h, 37 °C, 750 rpm), followed by trimethylsilylation with 20 µL BSTFA + 1 % TMCS (1 h, 37 °C, 750 rpm). Metabolite profiles were acquired on an Agilent 7890A Gas Chromatograph coupled to a 5975C Mass Selective Detector, where 1 µL of derivatised sample was injected into a split/splitless inlet set at 250 °C. Chromatographic separation was achieved using a J&W scientific VF-5 ms capillary column (30 m × 0.25 mm × 0.25 µM + 10 m duraguard). Oven conditions were set at 35 °C starting temperature, held for 2 min, then ramped at 25 °C/min to 325 °C and held for 5 min. Helium was used as the carrier gas at a flow rate of 1 mL/min. Compounds were fragmented by electron impact (EI) ionization and detected across a m/z range of 50–600 amu, with a scan speed of 9.2 scans/s. The metabolomics data was analysed using a targeted and an untargeted approach. For the untargeted profiling, chromatograms were processed using PyMS (O’Callaghan et al. 2012) to align metabolites and quantify a representative target ion, and subsequently generate a data matrix. For targeted profiling, chromatograms were processed using Agilent’s Mass Hunter Quantitative Analysis software, where target ion areas for polar metabolites contained within the in-house Metabolomics Australia (MA_25C) metabolite library were integrated and output as a data matrix for downstream data analysis. Each detected metabolite was visually inspected and manually integrated if required. This resulted in a highly curated matrix representing the detected metabolites in each sample.

### Data analysis

Animals weight and blood glucose was compared between groups using Kruskal–Wallis analysis using MatLab, MathWorks.

#### Lipidomics

Peak integration of both internal standards and serum samples was performed using the MultiQuant™, Sciex, software with manual inspection as required. Relative concentrations of the lipids were calculated automatically based on the internal standards. Lipids with relative concentrations not exceeding three times the background noise in five or more of the analyzed samples were excluded for further analysis.

#### Normalization of lipid data

Serum lipids were normalized to total content of PC (relative concentration of individual lipid specie/relative concentration of total PC) within the sample to allow for differences in hydration level of individual mice. This resulted in decreased variance within mouse groups and improved statistical power. We use phosphatidylcholine (the most abundant phospholipid class) as an internal reference to assess the relative differences in plasma composition between mouse strains.

#### Statistical analysis

Serum lipid relative quantities were analyzed, individually and divided into lipid classes, using Kruskal–Wallis analysis. *p* values were corrected for multiple comparisons using the Benjamini–Hochberg method. *p* values were considered significant <0.05 and all statistical analysis were performed in SPSS from IBM, MatLab or R.

#### Polar metabolomics

After median normalization of the targeted metabolomics data, the serum samples from the three groups were analyzed by Kruskal–Wallis test using the Benjamini–Hochberg method to correct for multiple testing. Post-hoc, pair wise analyses were performed using the Mann–Whitney U test, and the resultant pair wise *p* values were corrected by the Dunn–Sidak approach using MatLab. The untargeted data was statistically analysed similarly.

## Results and discussion

### Characteristics of the animals

The three different groups of animals were well matched regarding age (all were 12 weeks), but varied in weight and blood glucose (Table 1). The RIP CD154 × RAG KO mice had higher blood glucose levels compared to the RAG KO and control mice. This is in agreement with previous reports, where significant fluctuations in blood glucose over a time period of 40 weeks was recorded in this model (Haase et al. 2004; Haase and Markholst 2007). The authors explained this by insufficient ability of the beta-cells to produce the required insulin amounts to preserve blood glucose homeostasis, because of the local DC infiltration of the islets.

**Table 1:** Percentage change of weight, blood glucose and lipids by class from control C57BL/6

	RIP CD 154 × RAG KO (n = 6) change in % from control	RAG KO (n = 3) change in % from control
Weight*	3.5	11.8
BG serum*	102.67	6.69
Dihydroceramide (dhCer)*^,#^	77.78	77.78
Ceramide (Cer)*^,#^	29.22	22.73
Monohexosylceramide (MHC)	20.84	38.41
Dihexosylceramide (DHC)**^,#^	50	37.5
Trihexosylceramide (THC)	0	50
GM3 ganglioside (GM3)	−18.18	9.09
Alkylphosphatidylcholine (PC(O))	−8.15	−7.45
Sphingomyelin (SM)	−0.32	16.53
Alkenylphosphatidylcholine (plasmalogen, PC(P))	0.19	14.53
Lysophosphatidylcholine (LPC)**^,#^	−15.39	−4.16
Lysoalkylphosphatidylcholine (lysoplatelet activating factor, LPC(O))	−12.35	8.64
Phosphatidylethanolamine (PE)	9.02	−11.22
Alkylphosphatidylethanolamine (PE(O))*^,#^	28.42	40.7
Alkenylphosphatidylethanolamine (plasmalogen PE(P))*	16.77	35.98
Lysophosphatidylethanolamine (LPE)^#^	13.72	21.27
Phosphatidylinositol (PI)	2.43	16.79
Lysophosphatidylinositol (LPI)**^,#^	26.02	58.86
Phosphatidylserine (PS)	−28.89	−6.11
Phosphatidylglycerol (PG)**^,#,$^	−33.33	−50
Free cholesterol (COH)*	12.11	28.38
Cholesteryl ester (CE)**^,#^	−19.73	−9.5
Diacylglycerol (DG)	22.98	−13.13
Triacylglycerol (TG)	−7.45	−49.6

* *p* < 0.05 in the KW comparison

** *p* < 0.05 after Benjamini–Hochberg correction

^#^ *p* < 0.05 in RIP CD154 × RAG KO versus control

^$^ *p* < 0.05 in RAG KO versus control

### Lipid measurements

#### Reproducibility of the assay

To assess the assay performance of the entire procedure six quality control (QC) human plasma pool samples were evenly integrated in the extraction process amongst the serum samples from the animals, and were analyzed accordingly with the MRM method. The median intra-run coefficient of variation (% CV) was 5.88 % (supplementary Table 1).

#### Identification and quantification of lipids in serum

A total of 351 lipids from 21 different lipid classes and subclasses were identified in the serum of the murine models and relative quantities of these were compared between the RIP CD 154 × RAG KO, RAG KO and control mice (supplementary Table 1). We compared the RIP CD154 × RAG KO mice to RAG KO mice also to identify early metabolomic alterations, without the immune component, as we anticipated that the RAG KO model would be the best suited control in regards to the transgene element of the model.

Among the most striking differences in the overall lipid classes and subclasses were the LPCs, DHCs, LPI, PG and CEs (compared by ANOVA, *p* < 0.05 after Benjamini–Hochberg correction, Table 1). The LPCs were present in lower amounts in the RIP CD154 × RAG KO and RAG KO mice compared to control with the lowest levels found in the RIP CD154 × RAG KO. The shorter chain LPC’s (<18 C) were significantly reduced in the RIP CD154 × RAG KO mice compared to the control mice, but not RAG KO mice (sup Table 1). LPC differences in relation to progression to T1D have previously been identified in NOD mice (Sysi-Aho et al. 2011). In a subset of NOD mice negative for insulin antibodies (IAA) but later developing T1D, marginal higher levels of LPC were found as compared to NOD IAA negative animals that did not progress to T1D (Sysi-Aho et al. 2011). The latter group is comparable to our model in terms of lack of antibodies and non-progression to T1D and accordingly our results follows these prior observations (Sysi-Aho et al. 2011). Clinical research has also revealed changes in LPCs according to T1D progression. Oresic and coworkers identified transiently elevated overall serum LPC levels in children who later developed T1D (Orešič et al. 2008) and a 1.3–1.5 fold increase in the LPC (18:0/0:0) prior to seroconversion. Our results revealed higher levels of the LPC 18:0 (sup Table 1) in the RIP CD154 × RAG KO mice compared to the RAG KO mice and control. A recent study indicates that the LPCs have dual functions; they are capable of activating TLR4 and TLR2-1 mediated proinflammatory signaling, but in the presence of classical TLR ligands, the LPCs counteract some of the TLR mediated responses and overall induce an anti inflammatory environment (Carneiro et al. 2013). Overall it seems reasonable to speculate that LPCs might have different roles in each stage of the T1D disease pathogenesis. Low levels of LPCs in childhood have been linked to the transport of choline to tissues (Croset et al. 2000), and choline deficiencies have been associated to the development of T1D (Orešič et al. 2008). Later in the disease process, LPC may act as immune modulators through the lysophosphatidylcholine receptor an immunoregulatory G protein-coupled receptor named G2A whose genetic ablation resulted in the development of inflammatory autoimmune disease (Kabarowski et al. 2002).

Total serum CE levels differed between the groups of mice, with the lowest level found in the RIPCD154 × RAG KO mice. Previous research in the esterification of cholesterol in type 1 diabetic patients has revealed a lower esterification percentage of cholesterol in the very low density lipoprotein (VLDL) and intermediate density lipoprotein (IDL) compared to that of control subjects and a general increase in cholesterol absorption (Gylling et al. 2004). The RIP CD154 × RAG KO mice had borderline significant higher free cholesterol (COH) levels compared to control, but the overall difference between groups were not significant after correction for multiple comparison. Previous research on CE synthesis in relation to atherosclerosis in T1D in humans revealed an accumulation of CE in macrophages incubated with low density lipoproteins (LDL) from T1D patients compared to LDL from control, and a parallel higher accumulation of CE in the T1D patients. The authors also found that the non-enzymatic glycosylation of LDL in the T1D patients were higher and correlated with the CE synthesis. The glycosylation is possibly explained by the higher blood glucose in T1D patients, and this may in general contribute to the acceleration of atherosclerosis seen in diabetic patients (Lyons et al. 1987). This is in contradiction to our results, but may be a consequence of the transgenic mice not being able to produce mature macrophages because of the knock out of the RAG genes.

Total levels of DHC varied considerably (*p* < 0.05) among the three different types of mice (Table 1) with lower levels detected in control mice. DHC as well as MHC and THC are metabolites of Cer and the precursor dhCer. The two transgenic models had generally higher levels of the three metabolites compared to control mice. In the RIP CD154 × RAG KO mice the dhCer and Cer levels were in general higher in comparison to the RAG KO mice but the metabolites of the ceramides were lower, except for DHC. This particular expression pattern of the ceramides and its metabolites has been observed previously in two cohorts of pre-type 2 diabetic patients, where up regulation of de novo ceramide synthesis were not reflected in the downstream metabolites of ceramide (Meikle et al. 2013). Studies on streptozotocin induced T1D mice also showed accumulations of ceramides in the muscles of the animals after one week of insulin deprivation (Zabielski et al. 2014). Ceramides are found in high concentrations within the cell membranes and the membranes of the organelles, and the composition of the ceramides has been suspected to affect the function of the organelles. Recent advances in technology has facilitated investigating the role of ceramides in beta-cell dysfunction, and the current debate on ceramides role in T1D focuses on whether ceramides can mimic the effects of IL-1β in promoting beta-cell death and in repressing insulin production, reviewed in the paper by Boslem et al. (Boslem et al. 2012).

Interestingly, we also detected differing levels of LPI and PG in the comparison of all three groups of mice. Significant higher levels of LPI and lower levels of PG were seen in RIP CD154 × RAG KO compared to control mice. LPI affects numerous functions such as cell growth, differentiation and motility in a number of different cell-types through the orphan G protein-coupled receptor GPR55 specific receptor for LPI (Piñeiro and Falasca 2012). The LPIs and their receptors have been implicated in both physiological and pathophysiological processes such as autoimmune diseases and inflammation (Kihara et al. 2015). PGs are primarily confined to the mitochondria where they support membrane structure and functions as a substrate for the synthesis of cardiolipin (Zhang et al. 2014b). Cardiolipin is an important component of the inner mitochondrial membrane, and is particularly susceptible to attacks of reactive oxygen species (ROS) leading to the formation of cardiolipin peroxidation that can activate apoptosis (Paradies et al. 2009). To our knowledge no previous studies have addressed the role of these lipids in diabetes; consequently future studies are needed to investigate their potential role in the early stages of T1D.

### Polar metabolomics

#### Reproducibility of the metabolomic analysis

The reproducibility of the method was assessed by incorporating nine pooled quality control samples throughout the analysis sequence. The median % CV was 17.85 of the analytes (Table 2). All metabolites with CV >20 % were inspected manually, and were identified as compounds found in very low abundance.

**Table 2:** Percentage change, and coefficient of variation, from control C57BL/6, of polar metabolites in serum

Biochemical pathway	Metabolite	% CV	RIP CD154 × RAG KO (n = 6) % change	RAG KO (n = 3) % change
Amino acids and derivatives	l-Alanine*^,#^	22.97	−47.37	−37.51
	l-Valine**^,#^	2.47	−37.28	−37.06
	l-Leucine*	4.6	−35.64	−34.27
	l-Isoleucine**^,#^	8.82	−35.06	−35.98
	l-Threonine**^,#^	3.87	−52.48	−41.79
	l-Proline**^,#^	7.93	−53.44	−43.30
	Glycine*	13.03	−23.76	−20.98
	l-Serine**^,#^	5.88	−41.17	−29.30
	Beta-alanine*	12.11	−11.24	−16.85
	DL-homoserine**^,#^	22.55	−57.14	−50.00
	l-Aspartic acid**^,#^	15.33	−50.54	−11.83
	Trans-4-hydroxyl-l-proline	17.86	−29.37	−27.78
	l-Methionine**^,#^	17.84	−66.9	−49.8
	Pyroglutamic acid**^,#^	17.61	−38.75	−24.78
	l-Phenylalanine**^,#^	23.08	−60.02	−46.77
	Taurine*	58.84	−41.34	−74.56
	l-Ornithine**^,#^	30.30	−71.73	−73.25
	l-Lysine**^,#^	21.94	−68.27	−48.48
	Adenine	46	−45.45	27.27
	l-Tyrosine**^,#^	10.07	−59.57	−60.80
	l-Tryptophan	20	−25.99	−33.55
	5-Oxoproline*	17.86	139.36	196.58
	Putrescine*^,#^	40.16	−46.15	−30.77
Citric acid cycle	Succinate	13.98	−33.62	−23.28
	Fumarate	5.43	−42.64	−27.13
	*Cis*-aconitate	21.22	−41.67	−25.00
	Citriate	2.92	−24.87	−24.37
	Isocitrate	4.6	−27.59	−26.44
	l-Malic acid	1.68	−41.02	−20.68
Carbohydrate	d-Erythrose	25.39	14.29	−42.86
	*Meso*-erythritol**^,$^	3.83	10.00	−26.67
	d-Ribose	15.36	−21.48	46.31
	d-Fructose**^,#^	43.5	287.44	−14.08
	d-Talose	138.08	116.54	−55.97
	Sucrose	5.55	−50.00	−19.57
	d-Maltose**^,#^	8.21	183.33	8.33
	Trehalose**^,#^	11.47	137.50	−18.75
Glycolysis	d-(-)-3-phosphoglyceric acid	43.22	−50.00	−25.00
	Fructose-6-phosphate	47.69	27.08	−25.00
	Glucose-6-phosphate	44.59	38.24	−30.88
	Uridine	21.3	9.76	7.32
Fatty acids	Tetradecanoic acid**^,#^	37.62	129.01	−35.50
	Hexadecanoic acid (palmitic acid)*	4.22	−12.85	−46.04
	Octadecanoic acid (stearic acid)*^,#^	47.69	54.15	3.56
Organic acid	Benzoic acid	5.24	1.85	−7.41
	Glyceric acid	40.22	3.42	−26.50
	*N*-acetyl-l-glutamic acid	29.9	0.00	−25.00
	d-Gluconic acid	32.40	15.38	−30.77
Neurotransmitter	Gamma-aminobutyric acid (GABA)*^,#^	25.04	−60.91	−2.73
Organic compound	1,6-Anhydro-beta-d-Glucose (Levoglucosan)*	15.24	16.67	−27.78
	Ribitol	18.47	−9.09	−27.27
	Glycerol-2-phosphate	33.73	−17.39	−34.78
	Pyridoxine	35.76	−10.00	−5.00
	Myo-Inositol*	0.7	34.37	39.51
	d-Ribose-5-phosphate	45.94	0.00	−25.00
	d-Myo-inositol-1-phosphate	10.52	−13.43	−41.79
Food addittive	d-Gluconic acid-delta-lactone	32.4	−22.22	8.33

Data are presented as percentage change from control. % CV refers to coefficient of variation in percent of the metabolite in the pooled serum quality control samples

* *p* < 0.05 before correction for multiple testing

** *p* < 0.05 after Benjamini–Hochberg correction

^#^ *p* < 0.05 in RIP CD154 × RAG KO versus control

^$^ *p* < 0.05 in RAG KO versus control

#### Identification and relative quantification of metabolites in serum

To determine if there were metabolic differences between these sample groups, a broad profiling metabolomics analysis was undertaken using the GC–MS to detect polar metabolites. Statistical analysis (PCA) of the 213 metabolites detected showed separation between the sample groups (not shown) and univariate analysis revealed that 78 of the 213 metabolites were significantly different between the group (*p* < 0.05 after Benjamini–Hochberg correction, supplementary Table 2). To explore the metabolomics data further, metabolites were subjected to database searches using in-house standards and NIST libraries which resulted in the identification of 56 metabolites of which 18 metabolites varied significantly *p* < 0.05 (after Benjamini–Hochberg correction) (Table 2), and were dominated by amino acids and their derivates and simple carbohydrates. 13 out of 23 amino acids and their derivatives varied significantly between the groups, with the lowest levels seen in the RIP CD154 × RAG KO mice, except for l-isoleucine, l-ornithine an l-tyrosine, where the lowest levels were observed in the RAG KO model (Table 2). Differences in the amino acid metabolites have been observed previously in children at risk for developing T1D, especially lower methionine levels were seen in children who developed autoantibodies at two years old compared to children who developed autoantibodies later in childhood or remained autoantibody negative (Pflueger et al. 2011). l-Methionine relative concentration was decreased in the RIP CD154 × RAG KO mice compared to RAG KO and control (Table 2). It is known that methionine is involved in numerous metabolic processes including the catabolic pathway of choline and serves as a precursor for other sulfur containing amino acids such as taurine (Pflueger et al. 2011). Taurine levels were decreased in RIP CD 154 × RAG KO and RAG KO compared to control, however not significantly after correcting for multiple comparisons (Table 2). Lower levels of taurine in plasma in T1D patients has previously been shown compared to control individuals (Franconi et al. 1995), and it is generally accepted that taurine has a cytoprotective function (Ito et al. 2012). Studies in pregnant wistar rats revealed that taurine supplementation to a low protein diet could reverse the effects of the low protein diet on the fetuses during gestation. The reduction in protein intake during pregnancy mainly targeted the beta-cells respiratory capacities in the fetuses, leaving the cells more vulnerable to high glucose levels and more sensitive to proapoptotic cytokines. Taurine supplementation of the maternal diet abolished the effect of the low protein diet in the fetuses, possibly through mediation of the mitochondrial metabolism (Reusens et al. 2008). The formation of taurine is based on methionine and involves a transmethylation. This reaction has previously been suggested to play a role in the T1D pathogenesis, because the turnover of methionine by transmethylation is fourfold higher than protein degradation and synthesis, and methionine is essential for lymphocyte proliferation (Pflueger et al. 2011). In our data, the l-methionine levels are lower in the transgene mice compared to control, but since the two transgenic models don’t produce lymphocytes, this relationship might not be relevant. In contrast to previous research, the branched chain amino acids, l-leucine, l-isoleucine and l-valine, were found in lower levels in the two genetically modified models, Oresic et al. found the branched chain amino acids increased before seroconversion in children progressing to T1D (Orešič et al. 2008).

Four out of eight simple carbohydrates differed significantly between the groups, with biggest differences seen in fructose (Table 2). We found the highest level of d-fructose in the RIP CD154 × RAG KO mice, and this is in agreement to an older study where higher levels of monosaccharides were detected in diagnosed T1D patients (Pitkänen 1996). The intermediates in both the glycolysis and the citric acid cycle along with several organic acids did not vary between the groups of mice.

## Conclusions

This profiling study provides further explanation to the metabolomic changes seen in the early stages of T1D. Many of our findings confirm previous results such as low LPC levels, accumulation of ceramides and methionine deficits in the development of T1D. Of special interest is the increased level of LPI and decreased level and PG seen in the RIP CD154 × RAK KO mice, both lipid classes involved in cell signaling and previously linked to autoimmune diseases. Further mechanistic studies are needed in these lipids to elucidate their potential role in the T1D pathogenesis.

## Electronic supplementary material

Relative quantities of all lipids Supplementary material 1 (DOCX 43 kb)

Relative quantities of un-annotated metabolites Supplementary material 2 (DOCX 31 kb)

Conditions for tandem mass spectrometry analysis of lipid species Supplementary material 3 (DOCX 18 kb)

## References

[CR1] American Diabetes Association (ADA) (2013). Economic costs of diabetes in the U.S. in 2012. Diabetes Care.

[CR2] Bennett SRM, Carbone FR, Karamalis F, Flavell RA, Miller JFAP, Heath WR (1998). Help for cytotoxic-T-cell responses is mediated by CD40 signalling. Nature.

[CR3] Bligh EG, Dyer WJ (1959). A rapid method of total lipid extraction and purification. Canadian Journal of Biochemistry and Physiology.

[CR4] Boslem E, Meikle PJ, Biden TJ (2012). Roles of ceramide and sphingolipids in pancreatic β-cell function and dysfunction. Islets.

[CR5] Carneiro AB, Iaciura BMF, Nohara LL, Lopes CD, Veas EMC, Mariano VS, Bozza PT, Lopes UG, Atella GC, Almeida IC, Silva-Neto MAC (2013). Lysophosphatidylcholine triggers TLR2- and TLR4-mediated signaling pathways but counteracts LPS-induced NO synthesis in peritoneal macrophages by inhibiting NF-κB translocation and MAPK/ERK phosphorylation. PLoS ONE.

[CR6] Croset M, Brossard N, Polette A, Lagarde M (2000). Characterization of plasma unsaturated lysophosphatidylcholines in human and rat. Journal of Biochemistry.

[CR7] Daneman D (2006). Type 1 diabetes. The Lancet.

[CR8] Eisenbarth GS (1986). Type I diabetes mellitus. New England Journal of Medicine.

[CR9] Franconi F, Bennardini F, Mattana A, Miceli M, Ciuti M, Mian M, Gironi A, Anichini R, Seghieri G (1995). Plasma and platelet taurine are reduced in subjects with insulin-dependent diabetes mellitus: effects of taurine supplementation. The American Journal of Clinical Nutrition.

[CR10] Grohmann U, Fallarino F, Silla S, Bianchi R, Belladonna ML, Vacca C, Micheletti A, Fioretti MC, Puccetti P (2001). CD40 ligation ablates the tolerogenic potential of lymphoid dendritic cells. The Journal of Immunology.

[CR11] Gylling H, Tuominen JA, Koivisto VA, Miettinen TA (2004). Cholesterol metabolism in type 1 diabetes. Diabetes.

[CR12] Haase C, Markholst H (2007). CD40 is required for development of islet inflammation in the RIP-CD154 transgenic mouse model of type 1 diabetes. Annals of the New York Academy of Sciences.

[CR13] Haase C, Skak K, Michelsen BK, Markholst H (2004). Local activation of dendritic cells leads to insulitis and development of insulin-dependent diabetes in transgenic mice expressing CD154 on the pancreatic β-cells. Diabetes.

[CR14] Hex N, Bartlett C, Wright D, Taylor M, Varley D (2012). Estimating the current and future costs of type 1 and type 2 diabetes in the UK, including direct health costs and indirect societal and productivity costs. Diabetic Medicine.

[CR15] IDF. (2013). Diabetes atlas. http://www.idf.org/diabetesatlas.

[CR16] Ito T, Schaffer S, Azuma J (2012). The potential usefulness of taurine on diabetes mellitus and its complications. Amino Acids.

[CR17] Kabarowski JHS, Xu Y, Witte ON (2002). Lysophosphatidylcholine as a ligand for immunoregulation. Biochemical Pharmacology.

[CR18] Kihara Y, Mizuno H, Chun J (2015). Lysophospholipid receptors in drug discovery. Experimental Cell Research..

[CR19] Lyons TJ, Klein RL, Baynes JW, Stevenson HC, Lopes-Virella MF (1987). Stimulation of cholesteryl ester synthesis in human monocyte-derived macrophages by low-density lipoproteins from type 1 (insulin-dependent) diabetic patients: the influence of non-enzymatic glycosylation of low-density lipoproteins. Diabetologia.

[CR20] Meikle PJ, Wong G, Barlow CK, Weir JM, Greeve MA, MacIntosh GL, Almasy L, Comuzzie AG, Mahaney MC, Kowalczyk A, Haviv I, Grantham N, Magliano DJ, Jowett JBM, Zimmet P, Curran JE, Blangero J, Shaw J (2013). Plasma lipid profiling shows similar associations with prediabetes and type 2 diabetes. PLoS ONE.

[CR21] Nerup J, Mandrap-Poulsen T, Helqvist S, Andersen HU, Pociot F, Reimers JI, Cuartero BG, Karlsen AE, Bjerre U, Lorenzen T (1994). On the pathogenesis of IDDM. Diabetologia.

[CR22] Nishana M, Raghavan SC (2012). Role of recombination activating genes in the generation of antigen receptor diversity and beyond. Immunology.

[CR23] O’Callaghan S, De Souza DP, Isaac A, Wang Q, Hodkinson L, Olshansky M, Erwin T, Appelbe B, Tull DL, Roessner U, Bacic A, McConville MJ, Likic VA (2012). PyMS: a Python toolkit for processing of gas chromatography-mass spectrometry (GC-MS) data. Application and comparative study of selected tools. BMC Bioinformatics.

[CR24] Orešič M, Simell S, Sysi-Aho M, Näntö-Salonen K, Seppänen-Laakso T, Parikka V, Katajamaa M, Hekkala A, Mattila I, Keskinen P, Yetukuri L, Reinikainen A, Lähde J, Suortti T, Hakalax J, Simell T, Hyöty H, Veijola R, Ilonen J, Lahesmaa R, Knip M, Simell O (2008). Dysregulation of lipid and amino acid metabolism precedes islet autoimmunity in children who later progress to type 1 diabetes. The Journal of Experimental Medicine.

[CR25] Paradies G, Petrosillo G, Paradies V, Ruggiero FM (2009). Role of cardiolipin peroxidation and Ca2+ in mitochondrial dysfunction and disease. Cell Calcium.

[CR26] Pflueger M, Seppänen-Laakso T, Suortti T, Hyötyläinen T, Achenbach P, Bonifacio E, Orešič M, Ziegler A-G (2011). Age- and islet autoimmunity-associated differences in amino acid and lipid metabolites in children at risk for type 1 diabetes. Diabetes.

[CR27] Piñeiro R, Falasca M (2012). Lysophosphatidylinositol signalling: New wine from an old bottle. Biochimica et Biophysica Acta (BBA)—Molecular and Cell Biology of Lipids.

[CR28] Pitkänen E (1996). Mannose, mannitol, fructose and 1,5-anhydroglucitol concentrations measured by gas chromatography/mass spectrometry in blood plasma of diabetic patients. Clinica Chimica Acta.

[CR29] Reusens B, Sparre T, Kalbe L, Bouckenooghe T, Theys N, Kruhøffer M, Ørntoft TF, Nerup J, Remacle C (2008). The intrauterine metabolic environment modulates the gene expression pattern in fetal rat islets: prevention by maternal taurine supplementation. Diabetologia.

[CR30] Ridge JP, Di Rosa F, Matzinger P (1998). A conditioned dendritic cell can be a temporal bridge between a CD4+ T-helper and a T-killer cell. Nature.

[CR31] Schoenberger SP, Toes REM, van der Voort EIH, Offringa R, Melief CJM (1998). T-cell help for cytotoxic T lymphocytes is mediated by CD40-CD40L interactions. Nature.

[CR32] Sysi-Aho M, Ermolov A, Gopalacharyulu PV, Tripathi A, Seppänen-Laakso T, Maukonen J, Mattila I, Ruohonen ST, Vähätalo L, Yetukuri L, Härkönen T, Lindfors E, Nikkilä J, Ilonen J, Simell O, Saarela M, Knip M, Kaski S, Savontaus E, Orešič M (2011). Metabolic regulation in progression to autoimmune diabetes. PLoS Computational Biology.

[CR33] Weir JM, Wong G, Barlow CK, Greeve MA, Kowalczyk A, Almasy L, Comuzzie AG, Mahaney MC, Jowett JBM, Shaw J, Curran JE, Blangero J, Meikle PJ (2013). Plasma lipid profiling in a large population-based cohort. Journal of Lipid Research.

[CR34] Zabielski P, Blachnio-Zabielska A, Lanza IR, Gopala S, Manjunatha S, Jakaitis DR, Persson XM, Gransee J, Klaus KA, Schimke JM, Jensen MD, Nair KS (2014). Impact of insulin deprivation and treatment on sphingolipid distribution in different muscle subcellular compartments of streptozotocin-diabetic C57Bl/6 mice. American Journal Physiology Endocrinology Metabolism.

[CR35] Zhang, A.-H., Qiu, S., Xu, H.-Y., Sun, H., & Wang, X.-J. (2014a). Metabolomics in diabetes. *Clinica Chimica Acta*, *429*, 106–110. 10.1016/j.cca.2013.11.037 24321733

[CR36] Zhang J, Xu D, Nie J, Han R, Zhai Y, Shi Y (2014). Comparative gene identification-58 (CGI-58) promotes autophagy as a putative lysophosphatidylglycerol acyltransferase. Journal of Biological Chemistry.

