# Supplementary material for: Lipidomic and metabolomic characterization of a genetically modified mouse model of the early stages of human type 1 diabetes pathogenesis
Source: Metabolomics. 2015 Nov 17;12:13. doi: 10.1007/s11306-015-0889-1 (PMC4648980; doi:10.1007/s11306-015-0889-1)
Supplement: Supplementary file 1 — Relative quantities of all lipids Supplementary material 1 (DOCX 43 kb) [file 11306_2015_889_MOESM1_ESM.docx]

Supplementary table 1: Percentage change of lipids from control C57BL/6, mean relative concentration and standard deviation of lipids

|  | RIP CD 154 x RAG KO | | | RAG KO | | |
| --- | --- | --- | --- | --- | --- | --- |
|  | Percentage | Mean (pmol/nmol PC) | (SD) | Percentage | Mean (pmol/nmol PC) | (SD) |
| dhCer 16:0 | 68.97 | 0.05 | 0.02 | 86.21 | 0.05 | 0.04 |
| dhCer 18:0 | 7.14 | 0.01 | 0.02 | -21.43 | 0.01 | 0.00 |
| dhCer 20:0 | 133.33 | 0.01 | 0.02 | 33.33 | 0.01 | 0.00 |
| dhCer 22:0** | 180.56 | 0.10 | 0.04 | 177.78 | 0.10 | 0.07 |
| dhCer 24:0**# | 93.85 | 0.13 | 0.04 | 86.15 | 0.12 | 0.07 |
| dhCer 24:1**# | -40.63 | 0.02 | 0.00 | -25.00 | 0.02 | 0.00 |
| Cer 16:0# | 60.38 | 0.26 | 0.09 | 25.79 | 0.20 | 0.10 |
| Cer 18:0 | -8.51 | 0.04 | 0.04 | -31.91 | 0.03 | 0.01 |
| Cer 20:0 | 44.00 | 0.04 | 0.03 | -8.00 | 0.02 | 0.01 |
| Cer 22:0**# | 96.83 | 0.43 | 0.06 | 83.71 | 0.41 | 0.16 |
| Cer 24:0**# | 70.66 | 1.04 | 0.14 | 60.33 | 0.98 | 0.31 |
| Cer 24:1**# | -62.99 | 0.18 | 0.05 | -47.82 | 0.25 | 0.01 |
| MHC 16:0 | 17.53 | 0.84 | 0.17 | 20.76 | 0.86 | 0.13 |
| MHC 18:0**# | -51.93 | 0.11 | 0.03 | -37.34 | 0.15 | 0.03 |
| MHC 20:0* | -27.43 | 0.13 | 0.02 | -0.57 | 0.17 | 0.03 |
| MHC 22:0**# | 102.76 | 2.05 | 0.35 | 116.39 | 2.19 | 0.60 |
| MHC 24:0**#$ | 87.55 | 1.34 | 0.17 | 93.99 | 1.39 | 0.30 |
| MHC 24:1**# | -51.65 | 0.69 | 0.16 | -19.11 | 1.15 | 0.14 |
| DHC 16:0*# | 45.16 | 0.04 | 0.01 | 38.71 | 0.04 | 0.01 |
| DHC 22:0**#$ | 136.36 | 0.03 | 0.00 | 127.27 | 0.02 | 0.01 |
| DHC 24:0 | 25.00 | 0.02 | 0.00 | 31.25 | 0.02 | 0.00 |
| DHC 24:1 | -3.85 | 0.03 | 0.00 | -7.69 | 0.02 | 0.00 |
| THC 16:0 | 0.00 | 0.02 | 0.01 | 41.18 | 0.02 | 0.00 |
| THC 24:1 | 0.00 | 0.01 | 0.00 | -14.29 | 0.01 | 0.00 |
| GM3 16:0 | -16.67 | 0.04 | 0.01 | -7.14 | 0.04 | 0.00 |
| GM3 18:0**# | -30.56 | 0.03 | 0.00 | -2.78 | 0.04 | 0.00 |
| GM3 22:0**#$ | 162.50 | 0.02 | 0.00 | 175.00 | 0.02 | 0.00 |
| GM3 24:1**# | -45.45 | 0.01 | 0.00 | -13.64 | 0.02 | 0.01 |
| SM 31:1 | -11.90 | 0.04 | 0.01 | 11.90 | 0.05 | 0.01 |
| SM 32:0 | -18.18 | 0.01 | 0.00 | 9.09 | 0.01 | 0.00 |
| SM 32:1** | -11.01 | 0.40 | 0.04 | 16.63 | 0.52 | 0.08 |
| SM 32:2**# | -22.86 | 0.03 | 0.00 | 11.43 | 0.04 | 0.00 |
| SM 33:1**$ | 14.31 | 2.04 | 0.20 | 44.56 | 2.58 | 0.60 |
| SM 34:0 | 3.15 | 1.05 | 0.07 | 16.85 | 1.19 | 0.13 |
| SM 34:1 | 1.29 | 31.93 | 1.83 | 23.11 | 38.81 | 4.37 |
| SM 34:2 | 3.49 | 3.74 | 0.14 | 29.46 | 4.68 | 0.49 |
| SM 34:3 | -4.76 | 0.02 | 0.00 | 23.81 | 0.03 | 0.00 |
| SM 35:1 | -8.71 | 0.48 | 0.09 | 10.80 | 0.58 | 0.03 |
| SM 35:2 | 8.11 | 0.08 | 0.01 | 29.73 | 0.10 | 0.02 |
| SM 36:1**# | -36.21 | 2.88 | 0.46 | -13.71 | 3.90 | 0.15 |
| SM 36:2 | -12.46 | 1.00 | 0.16 | 5.31 | 1.21 | 0.04 |
| SM 36:3**#$ | 82.84 | 0.31 | 0.04 | 97.04 | 0.33 | 0.11 |
| SM 37:2* | -20.00 | 0.16 | 0.03 | -4.00 | 0.19 | 0.02 |
| SM 38:1**# | -38.95 | 5.10 | 1.08 | -23.39 | 6.39 | 2.36 |
| SM 38:2 | -4.04 | 11.28 | 0.73 | 1.39 | 11.92 | 1.30 |
| SM 39:1**# | 27.20 | 1.44 | 0.21 | 31.60 | 1.49 | 0.17 |
| SM 41:1 | -12.42 | 3.79 | 0.54 | 11.73 | 4.84 | 0.73 |
| SM 41:2**# | -30.62 | 1.12 | 0.23 | 1.48 | 1.64 | 0.09 |
| SM 42:1**#$ | 82.23 | 11.44 | 0.76 | 76.46 | 11.08 | 2.23 |
| PC 28:0**# | -45.45 | 0.01 | 0.00 | -45.45 | 0.01 | 0.00 |
| PC 29:0**# | -50.00 | 0.00 | 0.00 | -33.33 | 0.00 | 0.00 |
| PC 30:0**#$ | -54.15 | 0.27 | 0.06 | -44.16 | 0.33 | 0.09 |
| PC 31:0**#$ | -49.37 | 0.12 | 0.03 | -34.31 | 0.16 | 0.04 |
| PC 31:1**# | -30.94 | 0.10 | 0.01 | -14.39 | 0.12 | 0.00 |
| PC 32:0**# | -42.77 | 6.74 | 0.46 | -27.73 | 8.52 | 2.85 |
| PC 32:1**#$ | -61.45 | 1.45 | 0.22 | -49.72 | 1.89 | 0.42 |
| PC 32:2**# | -27.59 | 0.59 | 0.12 | -8.42 | 0.75 | 0.08 |
| PC 32:3**#$ | 37.50 | 0.05 | 0.01 | 62.50 | 0.07 | 0.02 |
| PC 33:0**#$ | -42.69 | 0.24 | 0.04 | -25.71 | 0.32 | 0.05 |
| PC 33:1**#$ | -61.95 | 0.36 | 0.06 | -57.02 | 0.40 | 0.06 |
| PC 33:2 | -16.21 | 1.75 | 0.31 | 2.92 | 2.15 | 0.28 |
| PC 33:3 | -34.21 | 0.02 | 0.01 | 2.63 | 0.04 | 0.02 |
| PC 34:0** | -8.25 | 3.81 | 0.23 | 30.46 | 5.42 | 0.44 |
| PC 34:1**# | -52.89 | 42.31 | 2.20 | -53.77 | 41.52 | 7.85 |
| PC 34:2 | -0.76 | 239.96 | 9.71 | -1.28 | 238.69 | 16.87 |
| PC 34:3**#$ | -29.65 | 7.02 | 1.26 | -15.85 | 8.40 | 0.35 |
| PC 34:4 | -19.07 | 0.16 | 0.04 | 4.12 | 0.20 | 0.02 |
| PC 34:5 | -25.00 | 0.01 | 0.00 | 0.00 | 0.01 | 0.00 |
| PC 35:0** | -14.53 | 0.10 | 0.01 | 15.38 | 0.14 | 0.01 |
| PC 35:1**#$ | -56.63 | 0.65 | 0.09 | -54.43 | 0.68 | 0.13 |
| PC 35:2 | -2.18 | 7.57 | 1.04 | 10.41 | 8.55 | 0.84 |
| PC 35:3**# | -43.93 | 0.64 | 0.15 | -22.62 | 0.88 | 0.02 |
| PC 35:4 | -15.71 | 0.38 | 0.10 | -2.65 | 0.44 | 0.05 |
| PC 35:5 | 16.67 | 0.02 | 0.04 | -94.44 | 0.00 | 0.01 |
| PC 36:0**#$ | 107.64 | 1.25 | 0.15 | 153.49 | 1.53 | 0.49 |
| PC 36:1 | 17.70 | 26.28 | 4.11 | 15.42 | 25.77 | 3.98 |
| PC 36:2**#$ | 33.41 | 260.17 | 20.88 | 33.97 | 261.27 | 15.54 |
| PC 36:3**#$ | 22.87 | 88.50 | 8.93 | 26.55 | 91.15 | 10.20 |
| PC 36:4b | -10.87 | 61.94 | 10.14 | -11.64 | 61.40 | 5.88 |
| PC 36:5**#$ | -69.60 | 2.04 | 0.45 | -59.73 | 2.70 | 0.61 |
| PC 36:6**#$ | -63.85 | 0.08 | 0.02 | -52.58 | 0.10 | 0.00 |
| PC 37:4 | 3.90 | 2.59 | 0.48 | 7.19 | 2.67 | 0.07 |
| PC 37:5**# | -55.03 | 0.13 | 0.04 | -41.95 | 0.17 | 0.06 |
| PC 37:6**#$ | -58.14 | 0.13 | 0.07 | -58.47 | 0.13 | 0.02 |
| PC 38:2 | 50.81 | 5.46 | 1.36 | 18.44 | 4.29 | 0.76 |
| PC 38:3 | 4.99 | 23.77 | 5.50 | -9.91 | 20.40 | 2.38 |
| PC 38:4**# | 47.62 | 104.08 | 8.71 | 34.72 | 94.98 | 13.30 |
| PC 38:5 | -17.80 | 23.87 | 3.49 | -9.69 | 26.23 | 0.50 |
| PC 38:6a**#$ | 86.42 | 8.90 | 1.90 | 119.61 | 10.48 | 2.53 |
| PC 38:6b**#$ | -46.15 | 32.82 | 5.03 | -43.27 | 34.58 | 5.10 |
| PC 38:7**#$ | -52.49 | 0.92 | 0.22 | -43.55 | 1.10 | 0.05 |
| PC 39:5**#$ | -56.51 | 0.20 | 0.08 | -47.46 | 0.24 | 0.05 |
| PC 39:6**#$ | -43.69 | 1.00 | 0.30 | -38.47 | 1.10 | 0.12 |
| PC 39:7**#$ | -80.00 | 0.04 | 0.02 | -73.33 | 0.05 | 0.02 |
| PC 40:4 | 8.10 | 1.11 | 0.34 | -14.93 | 0.87 | 0.19 |
| PC 40:5**#$ | -26.03 | 6.55 | 0.58 | -31.27 | 6.08 | 0.37 |
| PC 40:6**#$ | -30.11 | 24.76 | 3.21 | -37.17 | 22.25 | 2.32 |
| PC 40:7**# | -35.19 | 6.05 | 1.25 | -24.94 | 7.01 | 0.81 |
| PC 40:8**$ | 35.62 | 3.04 | 0.64 | 68.93 | 3.79 | 0.70 |
| PC(O-32:0)**# | -35.70 | 0.49 | 0.07 | -19.69 | 0.61 | 0.05 |
| PC(O-32:1)**#$ | -37.80 | 0.08 | 0.02 | -26.77 | 0.09 | 0.01 |
| PC(O-32:2) | -13.51 | 0.03 | 0.01 | 13.51 | 0.04 | 0.00 |
| PC(O-34:1)**#$ | -61.95 | 0.36 | 0.06 | -57.02 | 0.40 | 0.06 |
| PC(O-34:2)**#$ | 26.58 | 1.50 | 0.29 | 36.46 | 1.62 | 0.17 |
| PC(O-34:3)** | -25.00 | 0.04 | 0.01 | 6.25 | 0.05 | 0.01 |
| PC(O-34:4) | -14.71 | 0.06 | 0.01 | -1.47 | 0.07 | 0.00 |
| PC(O-35:4)**$ | 16.13 | 0.04 | 0.01 | 29.03 | 0.04 | 0.00 |
| PC(O-36:0)**#$ | 89.19 | 0.07 | 0.01 | 145.95 | 0.09 | 0.01 |
| PC(O-36:1) | 9.57 | 0.23 | 0.03 | 16.27 | 0.24 | 0.04 |
| PC(O-36:2)** | 22.76 | 1.01 | 0.18 | 39.23 | 1.15 | 0.10 |
| PC(O-36:3)**#$ | 25.68 | 0.96 | 0.13 | 35.72 | 1.04 | 0.14 |
| PC(O-36:4) | -2.70 | 2.49 | 0.52 | -1.33 | 2.53 | 0.16 |
| PC(O-36:5) | -9.30 | 0.08 | 0.02 | -18.60 | 0.07 | 0.01 |
| PC(O-38:4) | 9.28 | 1.26 | 0.16 | 17.26 | 1.35 | 0.15 |
| PC(O-38:5) | 11.69 | 2.44 | 0.33 | 22.19 | 2.67 | 0.21 |
| PC(O-40:5) | 7.57 | 0.45 | 0.08 | 13.95 | 0.48 | 0.05 |
| PC(O-40:6) | -0.81 | 0.86 | 0.11 | 3.70 | 0.90 | 0.17 |
| PC(O-40:7) | -5.81 | 0.73 | 0.14 | 1.68 | 0.79 | 0.03 |
| PC(P-30:0)**#$ | -58.82 | 0.01 | 0.00 | -58.82 | 0.01 | 0.00 |
| PC(P-32:0) | -13.64 | 0.21 | 0.02 | 4.96 | 0.25 | 0.02 |
| PC(P-32:1)**# | -37.04 | 0.02 | 0.00 | -22.22 | 0.02 | 0.00 |
| PC(P-34:1)**# | -23.81 | 0.16 | 0.03 | -2.38 | 0.21 | 0.02 |
| PC(P-34:2) | 5.47 | 0.64 | 0.05 | 24.54 | 0.75 | 0.07 |
| PC(P-34:3)**# | -39.13 | 0.03 | 0.00 | -15.22 | 0.04 | 0.01 |
| PC(P-36:2)**#$ | 21.43 | 0.20 | 0.02 | 53.57 | 0.26 | 0.02 |
| PC(P-36:4) | -2.50 | 1.13 | 0.17 | 23.23 | 1.43 | 0.25 |
| PC(P-36:5) | 20.69 | 0.04 | 0.02 | 17.24 | 0.03 | 0.00 |
| PC(P-38:4)**#$ | 61.60 | 0.61 | 0.03 | 74.93 | 0.66 | 0.05 |
| PC(P-38:5) | -6.69 | 1.32 | 0.21 | -2.18 | 1.39 | 0.12 |
| PC(P-38:6) | -18.43 | 0.42 | 0.06 | -17.66 | 0.43 | 0.16 |
| PC(P-40:6) | 7.58 | 0.60 | 0.05 | 22.38 | 0.68 | 0.07 |
| LPC 14:0**# | -49.49 | 0.35 | 0.05 | -18.05 | 0.56 | 0.09 |
| LPC 15:0**# | -39.58 | 0.54 | 0.11 | -18.95 | 0.73 | 0.05 |
| LPC 16:0**#$ | -35.20 | 94.14 | 5.02 | -22.57 | 112.50 | 7.97 |
| LPC 16:1**#$ | -51.83 | 1.81 | 0.26 | -32.67 | 2.53 | 0.11 |
| LPC 17:0**# | -29.23 | 1.91 | 0.24 | -2.49 | 2.63 | 0.14 |
| LPC 17:1**#$ | -57.74 | 0.24 | 0.03 | -39.30 | 0.35 | 0.07 |
| LPC 18:0**# | 12.25 | 51.88 | 3.53 | 18.77 | 54.89 | 5.90 |
| LPC 18:1 | -9.55 | 26.00 | 1.75 | 6.46 | 30.60 | 3.79 |
| LPC 18:2 | 8.20 | 62.57 | 3.51 | 16.57 | 67.41 | 9.04 |
| LPC 18:3**# | -40.06 | 1.24 | 0.11 | -14.43 | 1.77 | 0.21 |
| LPC 20:0**#$ | 166.34 | 1.08 | 0.29 | 121.29 | 0.89 | 0.25 |
| LPC 20:1**#$ | 63.42 | 1.19 | 0.15 | 57.12 | 1.15 | 0.14 |
| LPC 20:2*$ | 11.35 | 1.05 | 0.23 | 62.78 | 1.54 | 0.41 |
| LPC 20:3**# | -20.26 | 6.62 | 0.92 | -3.84 | 7.98 | 1.26 |
| LPC 20:4**# | 14.60 | 30.10 | 2.87 | 25.47 | 32.96 | 5.33 |
| LPC 20:5**#$ | -86.89 | 0.23 | 0.10 | -79.56 | 0.35 | 0.17 |
| LPC 22:0**#$ | 118.24 | 0.37 | 0.08 | 90.00 | 0.32 | 0.08 |
| LPC 22:1**$ | 14.49 | 0.08 | 0.01 | 27.54 | 0.09 | 0.00 |
| LPC 22:5**#$ | -47.53 | 1.43 | 0.16 | -40.94 | 1.60 | 0.15 |
| LPC 22:6**# | -32.68 | 14.53 | 2.29 | -25.75 | 16.03 | 0.99 |
| LPC 24:0**#$ | 52.84 | 0.46 | 0.07 | 52.84 | 0.46 | 0.10 |
| LPC(O-16:0)**# | -29.74 | 0.27 | 0.04 | -16.67 | 0.33 | 0.02 |
| LPC(O-18:0) | -1.92 | 0.10 | 0.01 | 19.23 | 0.12 | 0.01 |
| LPC(O-18:1) | -7.31 | 0.20 | 0.02 | 20.55 | 0.26 | 0.04 |
| LPC(O-20:0)**$ | 16.67 | 0.05 | 0.01 | 52.38 | 0.06 | 0.00 |
| LPC(O-20:1)**$ | 14.29 | 0.01 | 0.00 | 57.14 | 0.01 | 0.00 |
| LPC(O-22:0)**#$ | 55.56 | 0.03 | 0.00 | 83.33 | 0.03 | 0.01 |
| LPC(O-22:1)**#$ | 37.50 | 0.01 | 0.00 | 87.50 | 0.02 | 0.00 |
| LPC(O-24:0)**#$ | 137.50 | 0.02 | 0.00 | 150.00 | 0.02 | 0.00 |
| LPC(O-24:1)**#$ | 40.00 | 0.01 | 0.00 | 90.00 | 0.02 | 0.00 |
| LPC(O-24:2)**$ | 50.00 | 0.00 | 0.00 | 100.00 | 0.00 | 0.00 |
| PE 32:0**# | -53.13 | 0.01 | 0.00 | -18.75 | 0.03 | 0.01 |
| PE 34:1**# | -38.07 | 0.13 | 0.03 | -28.90 | 0.15 | 0.04 |
| PE 34:2 | 2.52 | 0.81 | 0.29 | -15.99 | 0.67 | 0.14 |
| PE 34:3**#$ | -57.58 | 0.01 | 0.01 | -60.61 | 0.01 | 0.01 |
| PE 35:1**#$ | 69.44 | 0.12 | 0.01 | 84.72 | 0.13 | 0.02 |
| PE 35:2*$ | -25.00 | 0.04 | 0.02 | -36.54 | 0.03 | 0.01 |
| PE 36:0 | -11.11 | 0.02 | 0.00 | -3.70 | 0.03 | 0.01 |
| PE 36:1**#$ | 115.94 | 0.60 | 0.20 | 54.71 | 0.43 | 0.12 |
| PE 36:2**# | 82.79 | 4.76 | 1.80 | 37.73 | 3.58 | 1.22 |
| PE 36:3*# | 54.02 | 1.07 | 0.39 | 24.43 | 0.87 | 0.26 |
| PE 36:4**#$ | -34.61 | 0.70 | 0.18 | -37.97 | 0.67 | 0.07 |
| PE 36:5**#$ | -67.74 | 0.02 | 0.01 | -53.23 | 0.03 | 0.01 |
| PE 38:3 | -1.12 | 0.44 | 0.10 | -19.55 | 0.36 | 0.02 |
| PE 38:4 | 10.34 | 2.70 | 0.76 | -11.98 | 2.15 | 0.18 |
| PE 38:5**# | -35.21 | 0.67 | 0.17 | -29.18 | 0.73 | 0.11 |
| PE 38:6**#$ | -46.23 | 0.89 | 0.27 | -56.97 | 0.71 | 0.05 |
| PE 40:4 | 16.67 | 0.04 | 0.01 | 30.56 | 0.05 | 0.03 |
| PE 40:5 | 20.00 | 0.02 | 0.00 | 0.00 | 0.02 | 0.01 |
| PE 40:6**$ | -23.58 | 0.61 | 0.18 | -47.41 | 0.42 | 0.02 |
| PE 40:7**# | -41.60 | 0.23 | 0.06 | -33.33 | 0.27 | 0.07 |
| PE(O-34:1)**#$ | 70.97 | 0.11 | 0.02 | 93.55 | 0.12 | 0.03 |
| PE(O-34:2)**#$ | 41.30 | 0.06 | 0.01 | 91.30 | 0.09 | 0.02 |
| PE(O-36:2)**#$ | 69.85 | 1.14 | 0.13 | 90.45 | 1.28 | 0.26 |
| PE(O-36:3) | -21.10 | 0.09 | 0.02 | 0.92 | 0.11 | 0.01 |
| PE(O-36:4) | -17.02 | 0.12 | 0.03 | -16.31 | 0.12 | 0.02 |
| PE(O-36:5)**#$ | -54.17 | 0.01 | 0.01 | -50.00 | 0.01 | 0.00 |
| PE(O-38:4)**# | 40.04 | 1.43 | 0.25 | 41.21 | 1.45 | 0.18 |
| PE(O-38:5) | -21.54 | 0.19 | 0.04 | -8.94 | 0.22 | 0.04 |
| PE(O-40:5) | -5.80 | 0.41 | 0.05 | 19.72 | 0.52 | 0.05 |
| PE(O-40:6) | -17.14 | 0.03 | 0.01 | -14.29 | 0.03 | 0.00 |
| PE(O-40:7) | 17.19 | 0.08 | 0.01 | 17.19 | 0.07 | 0.00 |
| PE(P-34:1) | 4.35 | 0.02 | 0.01 | 21.74 | 0.03 | 0.01 |
| PE(P-34:2) | -6.90 | 0.03 | 0.01 | 27.59 | 0.04 | 0.00 |
| PE(P-36:1)**#$ | 69.85 | 1.14 | 0.13 | 90.45 | 1.28 | 0.26 |
| PE(P-36:2) | -21.82 | 0.09 | 0.02 | -0.91 | 0.11 | 0.01 |
| PE(P-36:4) | -27.93 | 0.08 | 0.02 | -10.81 | 0.10 | 0.04 |
| PE(P-38:4)**#$ | 59.15 | 0.23 | 0.03 | 91.55 | 0.27 | 0.04 |
| PE(P-38:5) | -6.96 | 0.47 | 0.07 | 16.10 | 0.58 | 0.17 |
| PE(P-38:6) | -14.06 | 0.16 | 0.04 | -5.73 | 0.18 | 0.02 |
| PE(P-40:4)**#$ | 141.18 | 0.08 | 0.01 | 205.88 | 0.10 | 0.02 |
| PE(P-40:5) | 2.64 | 1.32 | 0.22 | 18.29 | 1.53 | 0.26 |
| PE(P-40:6)* | 21.26 | 0.21 | 0.03 | 42.53 | 0.25 | 0.05 |
| LPE 16:0**#$ | -47.57 | 2.40 | 0.42 | -38.68 | 2.81 | 0.07 |
| LPE 18:0 | 7.15 | 6.52 | 1.01 | 16.99 | 7.12 | 1.16 |
| LPE 18:1**# | 67.20 | 6.22 | 0.78 | 81.33 | 6.75 | 2.62 |
| LPE 18:2**# | 70.71 | 21.57 | 3.40 | 85.77 | 23.47 | 10.27 |
| LPE 20:4 | 0.96 | 7.58 | 1.20 | 6.71 | 8.01 | 1.93 |
| LPE 22:6**#$ | -34.14 | 6.93 | 1.49 | -38.58 | 6.46 | 1.06 |
| PI 32:0**# | -32.17 | 0.10 | 0.02 | -25.87 | 0.11 | 0.01 |
| PI 32:1**# | -62.16 | 0.03 | 0.02 | -39.19 | 0.05 | 0.01 |
| PI 34:0 | -14.13 | 0.08 | 0.02 | 3.26 | 0.09 | 0.06 |
| PI 34:1 | 11.60 | 1.60 | 0.35 | 7.27 | 1.54 | 0.37 |
| PI 36:1**# | 134.59 | 3.11 | 0.86 | 132.55 | 3.09 | 1.41 |
| PI 36:2**#$ | 79.91 | 30.30 | 6.90 | 114.82 | 36.18 | 13.28 |
| PI 36:3 | 16.49 | 8.60 | 1.93 | 47.01 | 10.85 | 3.09 |
| PI 36:4**#$ | -57.18 | 7.15 | 1.21 | -45.82 | 9.05 | 1.98 |
| PI 38:2 | -29.97 | 0.21 | 0.07 | -28.34 | 0.22 | 0.05 |
| PI 38:3 | -18.20 | 3.58 | 1.11 | -5.92 | 4.12 | 0.48 |
| PI 38:4 | 0.17 | 67.82 | 12.21 | 9.28 | 73.98 | 10.11 |
| PI 38:5 | -20.67 | 3.88 | 1.09 | -3.72 | 4.70 | 0.36 |
| PI 38:6**#$ | -68.37 | 0.40 | 0.09 | -60.72 | 0.49 | 0.06 |
| PI 40:4 | -15.72 | 0.25 | 0.05 | -7.36 | 0.28 | 0.06 |
| PI 40:5**#$ | -54.80 | 0.30 | 0.08 | -48.05 | 0.35 | 0.05 |
| PI 40:6**#$ | -53.81 | 0.75 | 0.18 | -36.78 | 1.03 | 0.19 |
| LPI 18:0 | -1.78 | 0.83 | 0.09 | -3.80 | 0.81 | 0.15 |
| LPI 18:1**#$ | 107.90 | 0.60 | 0.13 | 125.09 | 0.66 | 0.26 |
| LPI 18:2**#$ | 81.16 | 4.13 | 1.15 | 149.91 | 5.70 | 2.62 |
| LPI 20:4 | 2.60 | 5.53 | 0.92 | 26.42 | 6.81 | 1.38 |
| PS 36:1 | -20.48 | 0.13 | 0.03 | 6.02 | 0.18 | 0.13 |
| PS 36:2 | -14.73 | 0.19 | 0.05 | 4.02 | 0.23 | 0.15 |
| PS 38:3 | -31.45 | 0.11 | 0.04 | -16.98 | 0.13 | 0.10 |
| PS 38:4 | -14.77 | 0.48 | 0.10 | 12.10 | 0.63 | 0.42 |
| PS 38:5**# | -44.20 | 0.08 | 0.02 | -7.97 | 0.13 | 0.04 |
| PS 40:5*# | -43.57 | 0.08 | 0.03 | -20.00 | 0.11 | 0.07 |
| PS 40:6**# | -47.33 | 0.22 | 0.05 | -30.83 | 0.28 | 0.19 |
| PG 36:2**#$ | -42.62 | 0.04 | 0.01 | -50.82 | 0.03 | 0.01 |
| COH* | 12.11 | 391.48 | 33.35 | 28.38 | 448.28 | 79.44 |
| CE 14:0**#$ | -49.60 | 1.87 | 0.46 | -39.76 | 2.24 | 0.24 |
| CE 15:0**# | -46.05 | 1.90 | 0.42 | -23.62 | 2.69 | 0.67 |
| CE 16:0**# | -37.05 | 54.49 | 3.98 | -16.77 | 72.04 | 15.26 |
| CE 16:1**#$ | -77.35 | 7.13 | 1.88 | -69.73 | 9.52 | 1.61 |
| CE 16:2**# | 40.22 | 0.78 | 0.25 | 77.56 | 0.99 | 0.34 |
| CE 17:0**#$ | -71.84 | 0.52 | 0.17 | -57.46 | 0.78 | 0.09 |
| CE 17:1**#$ | -50.15 | 3.42 | 0.30 | -29.50 | 4.84 | 0.78 |
| CE 18:0**# | -39.97 | 2.97 | 0.59 | -29.65 | 3.48 | 0.86 |
| CE 18:1**# | -24.54 | 48.62 | 7.62 | -0.99 | 63.79 | 5.33 |
| CE 18:2 | 1.51 | 109.91 | 15.29 | 7.88 | 116.81 | 11.42 |
| CE 18:3**#$ | -49.76 | 3.35 | 0.47 | -38.62 | 4.10 | 0.29 |
| CE 20:1 | -26.24 | 0.10 | 0.03 | -16.31 | 0.12 | 0.01 |
| CE 20:2**#$ | -45.65 | 0.28 | 0.03 | -36.56 | 0.33 | 0.01 |
| CE 20:3 | -1.64 | 16.01 | 2.34 | 0.41 | 16.35 | 2.84 |
| CE 20:4 | 7.61 | 101.17 | 16.03 | 7.86 | 101.40 | 9.42 |
| CE 20:5**#$ | -82.03 | 1.53 | 0.45 | -75.56 | 2.08 | 0.75 |
| CE 22:0*# | 76.00 | 0.04 | 0.03 | -8.00 | 0.02 | 0.01 |
| CE 22:1 | 22.73 | 0.03 | 0.03 | -36.36 | 0.01 | 0.00 |
| CE 22:4**#$ | -26.19 | 0.03 | 0.01 | -21.43 | 0.03 | 0.00 |
| CE 22:5**#$ | -38.94 | 0.90 | 0.14 | -38.87 | 0.90 | 0.15 |
| CE 22:6**#$ | -28.47 | 15.98 | 3.07 | -29.28 | 15.80 | 1.20 |
| CE 24:0 | 50.00 | 0.03 | 0.03 | -20.00 | 0.02 | 0.01 |
| CE 24:1**$ | -28.57 | 0.01 | 0.01 | -35.71 | 0.01 | 0.00 |
| CE 24:4 | 20.00 | 0.01 | 0.00 | 60.00 | 0.01 | 0.00 |
| CE 24:5**# | -69.23 | 0.00 | 0.00 | -53.85 | 0.01 | 0.00 |
| CE 24:6**#$ | -69.23 | 0.00 | 0.00 | -69.23 | 0.00 | 0.00 |
| DG 14:0/14:0 | 5.88 | 0.02 | 0.00 | 11.76 | 0.02 | 0.01 |
| DG 14:0/16:0 | -16.36 | 0.14 | 0.03 | -20.61 | 0.13 | 0.01 |
| DG 14:0/18:1**#$ | -32.47 | 0.05 | 0.01 | -44.16 | 0.04 | 0.01 |
| DG 14:0/18:2** | -30.99 | 0.05 | 0.02 | -54.93 | 0.03 | 0.02 |
| DG 16:0/16:0*# | -17.51 | 1.39 | 0.11 | -11.36 | 1.50 | 0.18 |
| DG 16:0/18:0 | -6.92 | 1.68 | 0.13 | -1.00 | 1.79 | 0.21 |
| DG 16:0/18:1**$ | -25.32 | 1.68 | 0.56 | -54.64 | 1.02 | 0.54 |
| DG 16:0/18:2 | 2.11 | 3.05 | 1.23 | -33.47 | 1.99 | 1.25 |
| DG 16:0/20:0 | -4.69 | 0.06 | 0.02 | -15.63 | 0.05 | 0.03 |
| DG 16:0/20:4**#$ | -49.50 | 0.10 | 0.02 | -57.92 | 0.08 | 0.03 |
| DG 16:0/22:6**#$ | -72.76 | 0.07 | 0.03 | -86.57 | 0.04 | 0.01 |
| DG 16:1/18:1**#$ | -58.89 | 0.19 | 0.05 | -64.67 | 0.17 | 0.04 |
| DG 18:0/18:0 | -4.76 | 1.24 | 0.14 | 2.00 | 1.33 | 0.16 |
| DG 18:0/18:1 | 22.45 | 0.91 | 0.33 | -17.88 | 0.61 | 0.45 |
| DG 18:0/18:2*# | 88.27 | 1.70 | 0.70 | 32.41 | 1.20 | 0.96 |
| DG 18:0/20:4 | -20.69 | 0.09 | 0.02 | -13.79 | 0.10 | 0.03 |
| DG 18:1/18:1 | 5.15 | 2.86 | 1.25 | -47.08 | 1.44 | 0.74 |
| DG 18:1/18:2 | 72.60 | 8.03 | 4.02 | -0.49 | 4.63 | 3.17 |
| DG 18:1/18:3 | 22.50 | 0.93 | 0.46 | -10.92 | 0.68 | 0.43 |
| DG 18:1/20:0**# | 263.16 | 0.14 | 0.10 | -21.05 | 0.03 | 0.02 |
| DG 18:1/20:3* | -50.20 | 0.12 | 0.05 | -55.02 | 0.11 | 0.05 |
| DG 18:1/20:4 | -4.33 | 0.97 | 0.20 | -17.73 | 0.83 | 0.33 |
| DG 18:2/18:2*# | 130.73 | 4.84 | 2.65 | 71.80 | 3.61 | 2.63 |
| TG 14:0/16:0/18:1**#$ | -74.00 | 0.11 | 0.03 | -84.63 | 0.07 | 0.01 |
| TG 14:0/16:0/18:2**#$ | -75.94 | 0.15 | 0.06 | -86.25 | 0.08 | 0.02 |
| TG 14:0/16:1/18:1**#$ | -71.14 | 0.07 | 0.02 | -80.49 | 0.05 | 0.01 |
| TG 14:0/16:1/18:2**$ | -57.75 | 0.06 | 0.03 | -82.39 | 0.02 | 0.01 |
| TG 14:0/17:0/18:1**#$ | -65.45 | 0.13 | 0.04 | -85.34 | 0.06 | 0.02 |
| TG 14:0/18:0/18:1**#$ | -56.25 | 0.02 | 0.01 | -79.17 | 0.01 | 0.00 |
| TG 14:0/18:2/18:2 | -23.13 | 0.23 | 0.16 | -61.56 | 0.11 | 0.09 |
| TG 14:1/16:0/18:1**$ | -53.57 | 0.03 | 0.01 | -73.21 | 0.01 | 0.00 |
| TG 14:1/16:1/18:0**#$ | -81.68 | 0.10 | 0.02 | -81.87 | 0.10 | 0.02 |
| TG 14:1/18:0/18:2 | -13.04 | 0.02 | 0.01 | -56.52 | 0.01 | 0.00 |
| TG 14:1/18:1/18:1**#$ | -59.36 | 0.24 | 0.12 | -79.93 | 0.12 | 0.04 |
| TG 15:0/16:0/18:1**#$ | -66.26 | 0.08 | 0.03 | -85.37 | 0.04 | 0.01 |
| TG 15:0/18:1/18:1**#$ | -50.77 | 0.10 | 0.04 | -80.00 | 0.04 | 0.03 |
| TG 16:0/16:0/16:0**#$ | -79.21 | 0.25 | 0.10 | -89.03 | 0.13 | 0.02 |
| TG 16:0/16:0/18:0**#$ | -70.47 | 0.40 | 0.18 | -84.68 | 0.21 | 0.07 |
| TG 16:0/16:0/18:1**#$ | -54.58 | 2.53 | 1.29 | -82.25 | 0.99 | 0.35 |
| TG 16:0/16:0/18:2**#$ | -54.84 | 2.71 | 1.31 | -80.71 | 1.16 | 0.63 |
| TG 16:0/16:1/17:0**#$ | -70.43 | 0.16 | 0.05 | -86.51 | 0.07 | 0.03 |
| TG 16:0/16:1/18:1**#$ | -81.65 | 0.70 | 0.26 | -88.79 | 0.43 | 0.08 |
| TG 16:0/17:0/18:0**#$ | -84.48 | 0.02 | 0.01 | -89.66 | 0.01 | 0.01 |
| TG 16:0/17:0/18:1**#$ | -62.45 | 0.09 | 0.04 | -86.90 | 0.03 | 0.01 |
| TG 16:0/17:0/18:2**#$ | -78.49 | 0.21 | 0.10 | -90.03 | 0.09 | 0.04 |
| TG 16:0/18:0/18:1**$ | -35.25 | 3.83 | 1.71 | -78.12 | 1.30 | 0.33 |
| TG 16:0/18:1/18:1**#$ | -47.47 | 12.17 | 5.50 | -77.58 | 5.19 | 2.05 |
| TG 16:0/18:1/18:2 | -14.08 | 21.90 | 8.88 | -56.17 | 11.17 | 5.91 |
| TG 16:0/18:2/18:2 | 4.84 | 12.85 | 5.28 | -37.91 | 7.61 | 4.83 |
| TG 16:1/16:1/16:1**#$ | -71.43 | 0.02 | 0.01 | -78.57 | 0.02 | 0.00 |
| TG 16:1/16:1/18:0**#$ | -64.02 | 0.07 | 0.03 | -79.37 | 0.04 | 0.02 |
| TG 16:1/16:1/18:1**#$ | -78.90 | 0.28 | 0.16 | -88.96 | 0.14 | 0.04 |
| TG 16:1/17:0/18:1**#$ | -59.12 | 0.33 | 0.13 | -83.97 | 0.13 | 0.06 |
| TG 16:1/18:1/18:1**#$ | -69.66 | 0.68 | 0.24 | -81.66 | 0.41 | 0.09 |
| TG 16:1/18:1/18:2**#$ | -47.51 | 3.59 | 1.41 | -69.61 | 2.08 | 0.98 |
| TG 17:0/18:1/18:1**#$ | -47.29 | 0.51 | 0.22 | -75.23 | 0.24 | 0.10 |
| TG 18:0/18:0/18:0 | 35.48 | 0.13 | 0.09 | -56.99 | 0.04 | 0.02 |
| TG 18:0/18:0/18:1 | 25.84 | 0.90 | 0.51 | -55.62 | 0.32 | 0.19 |
| TG 18:0/18:1/18:1 | 16.17 | 4.10 | 2.08 | -54.85 | 1.59 | 0.89 |
| TG 18:0/18:2/18:2 | 54.33 | 4.40 | 2.27 | 0.35 | 2.86 | 1.99 |
| TG 18:1/18:1/18:1 | 43.04 | 9.74 | 5.23 | -41.18 | 4.01 | 1.94 |
| TG 18:1/18:1/18:2 | 88.64 | 17.23 | 8.04 | 9.56 | 10.01 | 6.52 |
| TG 18:1/18:1/20:4 | 9.27 | 1.58 | 0.32 | -7.89 | 1.33 | 0.41 |
| TG 18:1/18:1/22:6**#$ | -48.33 | 2.11 | 0.60 | -57.08 | 1.75 | 0.28 |
| TG 18:1/18:2/18:2 | 113.09 | 21.81 | 10.11 | 24.37 | 12.73 | 8.61 |
| TG 18:2/18:2/18:2 | 91.94 | 6.12 | 3.55 | 35.53 | 4.32 | 3.27 |
| TG 18:2/18:2/20:4**# | 131.85 | 2.30 | 0.86 | 143.45 | 2.42 | 1.26 |

*p<0.05 in the Kruskal-wallis before adjusting for multiple testing, **p<0.05 after benjamini-Hochberg correction, #p<0.05 in RIP CD 154 x RAG KO vs control and $p<0.05 in RAG KO vs control. Dihydroceramide (dhCer), ceramide (Cer), monohexosylceramide (MHC), dihexosylceramide (DHC), trihexosylceramide (THC), GM3 ganglioside (GM3), sphingomyelin (SM), phosphatidylcholine (PC), alkylphosphatidylcholine (PC(O)), alkenylphosphatidylcholine (plasmalogen, PC(P)), lysophosphatidylcholine (LPC), lysoalkylphosphatidylcholine (lysoplatelet activating factor, LPC(O)), phosphatidylethanolamine (PE), alkylphosphatidylethanolamine (PE(O)), alkenylphosphatidylethanolamine (plasmalogen (PE(P)), phosphatidylinositol (PI), lysophosphatidylinositol (LPI), phosphatidylserine (PS), phosphatidylglycerol (PG), free cholesterol (COH), cholesteryl ester (CE), diacylglycerol (DG) and triacylglycerol (TG).
