# Supplementary material for: Lipidomic and metabolomic characterization of a genetically modified mouse model of the early stages of human type 1 diabetes pathogenesis
Source: Metabolomics. 2015 Nov 17;12:13. doi: 10.1007/s11306-015-0889-1 (PMC4648980; doi:10.1007/s11306-015-0889-1)
Supplement: Supplementary file 2 — Relative quantities of un-annotated metabolites Supplementary material 2 (DOCX 31 kb) [file 11306_2015_889_MOESM2_ESM.docx]

Supplementary table 2: Untargeted approach, metabolites percentage change from control C57BL/6

| Sample  Accession number and retention time | | Tentative ID | RIP CD154 x RAG KO % change from control | RAG KO % change from control |
| --- | --- | --- | --- | --- |
| 75_55_11_378:73* | | | -37.70 | -72.68 |
| 87_58_87_380:24 | | | -15.03 | -5.23 |
| 191_57_37_381:40 | | | -5.38 | 16.49 |
| 119_79_96_383:99 | | | -13.58 | 45.68 |
| 174_151_70_385:56 | | | 80.41 | 48.32 |
| 207_191_17_387:02 | | | -13.49 | -4.50 |
| 174_114_40_388:78$ | | | -3.83 | -18.03 |
| 58_56_4_389:53 | | | -6.61 | -10.34 |
| 318_225_58_391:77*$ | | | -6.75 | -18.99 |
| 207_56_29_393:06 | | | -42.82 | -38.21 |
| 152_72_23_393:73**# | | | 23.08 | 1.65 |
| 117_72_52_394:58*# | | | -53.13 | -26.66 |
| 221_79_37_396:17 | | | 28.57 | -17.24 |
| 174_74_66_398:44**# | | | -59.47 | -40.04 |
| 117_75_14_401:26**# | | Lactate | -34.50 | -33.38 |
| 59_58_48_401:98 | | | -21.00 | 2.01 |
| 174_130_71_403:13 | | | 15.69 | 4.38 |
| 190_75_97_403:98 | | | 2.15 | -10.75 |
| 148_66_88_406:62 | | | 25.64 | 0.85 |
| 75_55_10_407:93 | | | -18.60 | -9.30 |
| 72_57_43_409:02 | | | 23.08 | 7.69 |
| 116_75_42_410:25 | | | -8.33 | -4.17 |
| 151_77_36_412:08 | | | 3.85 | -1.44 |
| 77_75_23_414:19 | | | -6.50 | -9.03 |
| 116_75_4_415:94*# | | Alanine | -46.55 | -36.97 |
| 174_128_29_417:47 | | | -7.32 | -24.39 |
| 85_84_67_418:79 | | | -3.89 | -5.00 |
| 245_72_60_420:86**#$ | | | -10.31 | -23.71 |
| 258_116_62_421:29 | | | 11.48 | 21.89 |
| 102_72_3_422:38 | | Glycine pk1 | -31.95 | -9.29 |
| 131_116_8_423:31 | | | -21.56 | -38.62 |
| 191_148_57_424:56 | | | -32.71 | 2.80 |
| 218_148_67_426:46$ | | | -11.83 | -55.62 |
| 130_75_26_427:82 | | | -0.17 | 3.15 |
| 133_59_30_429:65 | | | 6.40 | 11.31 |
| 191_103_19_429:81 | | | -39.41 | -31.97 |
| 119_89_94_431:56 | | | 41.56 | 27.27 |
| 117_75_47_434:84 | | | 52.32 | 9.07 |
| 86_75_22_436:40 | | | -86.11 | 608.89 |
| 221_74_36_438:54 | | | 3.42 | 10.96 |
| 130_75_36_439:52**#$ | | | -46.09 | -41.98 |
| 72_58_21_440:27 | | | -6.13 | -17.45 |
| 74_59_25_442:06 | | | -2.59 | -6.03 |
| 221_117_33_443:81* | | | 53.85 | 61.54 |
| 86_75_65_444:85 | | | -14.29 | -2.38 |
| 70_66_16_446:90**# | | | -55.83 | -43.50 |
| 74_72_19_447:99* | | | 18.16 | -50.20 |
| 189_89_94_448:61 | | | -32.43 | -21.62 |
| 70_55_89_450:46 | | | 7.27 | -23.64 |
| 117_74_60_450:84*$ | | | -58.53 | -96.48 |
| 281_57_32_452:72 | | | -5.00 | -30.00 |
| 189_72_75_453:46# | Urea (overload) | | -33.87 | -33.87 |
| 149_144_20_454:65*# | Valine | | -20.67 | -10.05 |
| 318_69_40_456:46 | | | -7.94 | 23.81 |
| 191_74_35_457:78 | | | 6.28 | -14.66 |
| 86_72_64_458:71 | | | -24.22 | -16.41 |
| 277_132_65_459:49 | | | 70.97 | 48.39 |
| 117_74_48_460:75 | | | 9.66 | -11.36 |
| 159_114_47_462:40 | | | 40.63 | 56.25 |
| 261_245_33_463:76 | | | 208.33 | -46.21 |
| 319_231_30_467:02 | | | -5.47 | -5.47 |
| 189_146_16_470:29 | | | -7.98 | -14.92 |
| 205_117_51_471:15 | | | 0.60 | -3.93 |
| 189_146_16_472:15*$ | | | 78.25 | 201.75 |
| 158_74_10_473:09 | | | -33.36 | -30.55 |
| 170_169_90_475:33 | | | -17.14 | -20.00 |
| 158_100_5_480:67**# | | Leucine | -35.43 | -37.67 |
| 117_75_38_482:01 | | | -17.97 | -24.88 |
| 142_74_3_484:75*# | | Proline | -54.35 | -44.57 |
| 190_149_10_485:32*# | | | -28.57 | -14.29 |
| 174_86_12_486:38 | | | -21.28 | -24.10 |
| 75_55_35_487:50 | | | -33.69 | -22.99 |
| 117_75_72_488:92 | | | 0.00 | -5.88 |
| 204_191_39_489:42 | | | -11.73 | -8.16 |
| 189_103_52_490:34 | | | 4.21 | -26.32 |
| 241_99_69_496:59 | | | -28.00 | 32.00 |
| 245_75_19_498:98 | | | -42.86 | -19.05 |
| 141_99_40_499:98 | | | -5.81 | -10.47 |
| 204_100_16_500:56*# | | Serine | -41.54 | -29.23 |
| 149_80_37_501:58**# | | | 88.37 | 58.14 |
| 223_75_94_504:18* | | | -23.33 | -26.67 |
| 221_174_68_505:61 | | | -9.09 | 36.36 |
| 218_117_73_508:9**# | | Threonine | -54.96 | -44.11 |
| 72_58_34_510:42* | | | 4.55 | 4.55 |
| 246_240_62_511:71 | | | -3.45 | -24.14 |
| 202_72_76_512:49 | | | -1.79 | -7.14 |
| 103_55_72_516:74 | | | -7.69 | -10.26 |
| 174_154_46_524:22 | | | -11.33 | -16.67 |
| 104_61_75_524:79*# | | | -38.60 | -22.81 |
| 104_75_71_526:92 | | | 73.33 | 80.00 |
| 174_172_46_527:60**# | | | 19.01 | -11.57 |
| 350_133_25_531:32 | | | 36.73 | 36.73 |
| 146_70_56_534:32 | | | 31.58 | -31.58 |
| 174_86_23_535:76* | | | -35.90 | -41.03 |
| 218_75_28_536:87 | | | -35.24 | -70.48 |
| 247_232_43_537:36 | | | -6.67 | 6.67 |
| 233_133_55_540:54 | | | -39.96 | -19.02 |
| 158_72_90_543:80 | | | -12.50 | -12.50 |
| 217_149_81_544:46 | | | 5.26 | 0.00 |
| 156_155_52_544:84 | | | 40.68 | -16.95 |
| 337_221_67_547:80*$ | | | -4.17 | -37.50 |
| 305_75_79_549:20*# | | | 67.57 | -32.43 |
| 232_100_32_550:88 | | | -48.28 | -3.45 |
| 176_128_41_554:0**# | | Methionine | -70.52 | -49.25 |
| 292_205_56_555:61 | | | -30.00 | -34.78 |
| 156_75_5_556:87**# | | 5-oxoprolie | -43.18 | -29.60 |
| 175_130_45_557:81 | | | -8.16 | -29.59 |
| 207_84_54_559:05 | | | -20.51 | -11.54 |
| 292_205_54_560:14 | | | 2.78 | -8.89 |
| 263_70_23_561:90 | | | -11.11 | -4.63 |
| 313_75_51_565:79 | | | -4.76 | -41.90 |
| 129_85_15_566:47 | | | -16.67 | -28.79 |
| 120_75_92_567:79 | | | -39.11 | -28.08 |
| 192_142_94_573:41 | | | -47.26 | -49.32 |
| 217_204_38_575:02**# | | | 269.32 | -5.68 |
| 83_55_85_577:28* | | | -41.67 | 12.50 |
| 211_188_53_579:11 | | | 3.23 | -4.84 |
| 246_128_28_580:06*# | | Glutamate | -55.88 | -35.29 |
| 149_146_54_581:69 | | | 10.61 | -59.09 |
| 191_75_7_584:00 | | | 9.45 | 30.35 |
| 218_192_82_586:96* | | Phenylalanine | -54.41 | -48.13 |
| 204_103_35_587:56 | | | 27.21 | -52.33 |
| 103_74_14_589:29 | | | -34.48 | -39.66 |
| 191_75_36_590:02 | | | -1.80 | -10.81 |
| 211_75_87_592:35*# | | | -38.78 | -30.61 |
| 103_74_14_593:46 | | | -50.65 | 75.97 |
| 156_116_72_595:51**# | | Asparagine | -66.04 | -56.60 |
| 221_207_38_596:57 | | | -10.53 | -42.11 |
| 191_103_23_597:58 | | | 6.90 | 3.45 |
| 326_174_40_599:21 | | | -30.30 | -62.63 |
| 217_103_80_601:43 | | | -5.88 | -9.80 |
| 304_217_54_605:49 | | | -29.67 | 26.37 |
| 305_217_23_606:58 | | | 48.78 | -43.90 |
| 117_100_6_608:60 | | | 33.33 | 22.22 |
| 205_192_78_614:49 | | | -22.22 | 0.00 |
| 231_217_51_616:25* | | | 41.94 | -48.39 |
| 357_299_87_617:27 | | | 5.56 | 18.25 |
| 70_68_6_618:12 | | | 20.00 | 6.67 |
| 174_142_21_620:66 | | | -33.87 | -32.26 |
| 292_103_76_621:66 | | | 9.52 | -33.33 |
| 171_100_71_622:38* | | | -37.78 | -66.67 |
| 231_205_98_629:52 | | | 8.33 | -4.17 |
| 245_55_72_632:23 | | | -15.19 | -12.66 |
| 273_75_13_632:84 | | | -26.45 | -26.34 |
| 142_100_7_634:40**# | | Ornithine | -71.93 | -71.93 |
| 217_191_29_636:26 | | | 18.22 | -44.92 |
| 319_117_94_638:29 | | | 8.93 | -25.00 |
| 157_103_85_641:48**# | | | 417.42 | 81.82 |
| 217_191_57_642:74* | | | -42.91 | -30.99 |
| 285_75_68_644:49*#$ | | | -42.02 | -55.25 |
| 103_74_11_645:12**# | | Fructose Pk1* | 401.47 | -15.00 |
| 174_156_12_646:77**# | | Putrescine | -54.23 | -38.56 |
| 103_74_10_647:77**# | | Fructose Pk2 | 359.46 | -7.70 |
| 205_160_53_649:40**# | | Mannose | 125.41 | 16.66 |
| 319_205_78_651:70**# | | Glucose Pk1 | 19.48 | -9.04 |
| 319_205_97_654:39* | | Mannose pk2 | -15.56 | -8.27 |
| 179_116_4_657:32 | | | -16.62 | -11.34 |
| 319_205_85_657:99**# | | Glucose Pk2 | 23.30 | -9.15 |
| 319_205_72_659:09 | | | -42.11 | -34.17 |
| 205_103_56_660:46*# | | | 27.59 | -40.23 |
| 174_156_32_661:07**#$ | | Lysine | -85.52 | -80.51 |
| 323_207_74_662:10 | | | -8.66 | -9.53 |
| 333_219_35_662:67 | | | -10.00 | -12.50 |
| 217_191_59_665:14 | | | 2014.29 | 423.81 |
| 218_179_9_666:58**# | | Tyrosine | -78.00 | -80.93 |
| 217_204_25_667:88**# | | | 114.85 | -2.05 |
| 204_191_42_669:44**#$ | | | 931.15 | 670.49 |
| 217_204_39_670:98**# | | | 46.36 | -28.18 |
| 217_204_85_672:78* | | | -82.01 | 73.02 |
| 217_191_15_674:81* | | | 84.78 | 32.61 |
| 191_75_24_676:46 | | | -21.19 | -2.54 |
| 217_205_60_677:64 | | | 13.04 | -21.74 |
| 103_75_56_678:19* | | | -36.54 | -44.23 |
| 217_133_28_679:49**#$ | | Inositol? | 458.70 | 130.43 |
| 205_103_59_680:81*$ | | | 27.59 | -40.23 |
| 204_103_9_682:23**# | | | 102.97 | -12.87 |
| 318_305_91_684:41 | | | 18.75 | 21.88 |
| 393_103_57_685:12 | | | -33.33 | -26.67 |
| 117_75_81_686:77 | | | -12.39 | -51.33 |
| 75_55_50_687:98**#$ | | C16:0 | -57.80 | -75.72 |
| 117_75_66_692:02 | | | -13.46 | -46.17 |
| 191_75_8_693:83 | | | 0.00 | -37.04 |
| 305_217_91_699:99* | | myo-Inositol | 34.83 | 40.06 |
| 441_221_28_701:68 | | | -53.85 | -58.33 |
| 434_245_72_703:30 | | | -14.29 | -14.29 |
| 202_75_11_705:36**#$ | | | -65.22 | -60.87 |
| 103_56_75_711:66 | | | 25.00 | -10.00 |
| 56_55_3_717:31 | | | 12.22 | -3.33 |
| 103_75_36_723:85*$ | | | -13.64 | -61.36 |
| 75_67_60_725:80 | | | 46.15 | 126.92 |
| 75_67_57_729:89*# | | C18:2 | 83.58 | 34.42 |
| 339_117_91_730:65 | | | 51.83 | -16.06 |
| 79_78_15_731:52**#$ | | | 300.00 | 204.82 |
| 89_74_25_733:32*# | | | 54.09 | -10.38 |
| 117_75_67_735:31 | | | 52.89 | 3.31 |
| 103_98_60_739:80 | | | 18.42 | -26.32 |
| 103_98_39_741:10* | | | 53.85 | -26.92 |
| 191_174_49_743:29 | | | -25.00 | -62.50 |
| 103_98_85_745:38* | | | 50.00 | -25.00 |
| 56_55_2_746:86* | | | 39.13 | -78.26 |
| 217_204_8_748:95 | | | 10.22 | -52.55 |
| 191_103_25_753:17 | | | -9.68 | 9.68 |
| 117_103_88_755:91* | | C18:0 | 53.85 | -20.51 |
| 75_67_66_763:30 | | | 43.59 | 5.13 |
| 318_315_70_767:23 | | | -40.00 | -50.00 |
| 103_75_24_779:33 | | | 15.52 | -17.24 |
| 171_143_62_780:00 | | | -3.13 | -31.25 |
| 290_103_35_781:10 | | | 37.50 | -12.50 |
| 103_74_24_784:01 | | | 20.87 | -15.65 |
| 290_205_44_793:32 | | | 25.00 | -7.14 |
| 79_75_78_800:33* | | | -35.29 | -52.94 |
| 361_217_51_805:91 | | | -46.15 | -23.08 |
| 192_191_31_809:57**# | | | -75.00 | -62.00 |

*p<0.05 in the unadjusted KW, **p<0.05 after Benjamini-Hochberg correction, #p<0.05 in the RIP CD154 x RAG KO vs B6 and $p<0.05 in the RAG KO vs control. The first three numbers of the analyte ID refers to analyte number, the last two are the retention time.
