# Supplementary material for: Lipidomic and metabolomic characterization of a genetically modified mouse model of the early stages of human type 1 diabetes pathogenesis
Source: Metabolomics. 2015 Nov 17;12:13. doi: 10.1007/s11306-015-0889-1 (PMC4648980; doi:10.1007/s11306-015-0889-1)
Supplement: Supplementary file 3 — Conditions for tandem mass spectrometry analysis of lipid species Supplementary material 3 (DOCX 18 kb) [file 11306_2015_889_MOESM3_ESM.docx]

Supplementary table 3: Conditions for tandem mass spectrometry analysis of lipid species

|  | | | | | | Voltage settings (V) | | | |
| --- | --- | --- | --- | --- | --- | --- | --- | --- | --- |
| Lipid class or subclass | No. Of species | Internal standard | Pmol^a^ | Q1  (Parent ion) | Q3  (Product Ion) | DP | EP | CollE | CXP |
| dhCer | 6 | dhCer 8:0 | 100 | [M+H]^+^ | m/z 284.3 | 90 | 30 | 28 | 10 |
| Cer | 6 | Cer 17:0 | 100 | [M+H]^+^ | m/z 264.3 | 50 | 10 | 35 | 12 |
| MHC | 6 | MHC 16:0 *d*_3_ | 50 | [M+H]^+^ | m/z 264.3 | 77 | 10 | 50 | 12 |
| DHC | 6 | DHC 16:0 *d*_3_ | 50 | [M+H]^+^ | m/z 264.3 | 100 | 10 | 65 | 12 |
| THC | 6 | THC 17:0 | 50 | [M+H]^+^ | m/z 264.3 | 130 | 10 | 73 | 12 |
| GM | 6 | THC 17:0 | 50 | [M+H]^+^ | m/z 264.3 | 155 | 10 | 105 | 16 |
| SM | 19 | SM 12:0 | 200 | [M+H]^+^ | m/z 184.1 | 65 | 10 | 35 | 12 |
| PC | 41 | PC 13:0/13:0 | 100 | [M+H]^+^ | m/z 184.1 | 100 | 10 | 45 | 11 |
| PC(O) | 18 | PC 13:0/13:0 | 100 | [M+H]^+^ | m/z 184.1 | 100 | 10 | 45 | 11 |
| PC(P) | 8 | PC 13:0/13:0 | 100 | [M+H]^+^ | m/z 184.1 | 100 | 10 | 45 | 11 |
| LPC | 21 | LPC 13:0 | 100 | [M+H]^+^ | m/z184.1 | 90 | 10 | 38 | 12 |
| LPC(O) | 6 | LPC 13:0 | 100 | [M+H]^+^ | m/z 104.0 | 90 | 10 | 42 | 5 |
| PE | 18 | PE 17:0/17:0 | 100 | [M+H]^+^ | NL, 141 Da | 80 | 10 | 31 | 7 |
| PE(O) | 12 | PE 17:0/17:0 | 100 | [M+H]^+^ | NL, 141 Da | 80 | 10 | 31 | 7 |
| PE(P) | 9 | PE 17:0/17:0 | 100 | [M+H]^+^ | NL, 141 Da | 80 | 10 | 31 | 7 |
| LPE | 6 | LPE 14:0 | 100 | [M+H]^+^ | NL, 141 Da | 80 | 10 | 31 | 7 |
| PI | 17 | PE 17:0/17:0 | 100 | [M+NH_4_]^+^ | NL, 277 Da | 51 | 10 | 43 | 14 |
| LPI | 4 | LPE 14:0 | 100 | [M+NH_4_]^+^ | NL, 277 Da | 80 | 10 | 31 | 7 |
| PS | 7 | PS 17:0/17:0 | 100 | [M+H]^+^ | NL, 185 Da | 86 | 10 | 29 | 16 |
| PG | 4 | PG 17:0/17:0 | 100 | [M+NH_4_]^+^ | NL, 189 Da | 60 | 10 | 25 | 12 |
| CE | 26 | CE 18:0 *d*_6_ | 1000 | [M+NH_4_]^+^ | m/z 369.3 | 30 | 10 | 20 | 12 |
| COH | 1 | COH *d*_7_ | 1000 | [M+NH_4_]^+^ | m/z 369.3 | 55 | 10 | 17 | 12 |
| DG | 21 | DG 15:0/15:0 | 200 | [M+NH_4_]^+^ | NL, NH_3_ + fatty acid | 55 | 10 | 30 | 22 |
| TG | 43 | TG 17:0/17:0/17:0 | 100 | [M+NH_4_]^+^ | NL, NH_3_ + fatty acid | 95 | 10 | 30 | 12 |

The Q3 (Product ion) corresponds to either a specific product ion or a specific neutral loss (NL); DP, declustering potential; EP, entrance potential; CollE, collision energy; CXP, collision cell exit potential. ^a^ Amount of internal standard per sample.
